# Drunkards

**Published:** 1873-08

**Authors:** 


### DRUNKARDS

are made in two ways—by habit or by inheritance ; the latter is the more hopeless form, because the appetite is more remorseless, more unappeasable—it is in fact a part of the nature of the unfortunate. Three fourths of the idiotic children in a Massachusetts asylum were born of parents one or both of whom drank liquor. But if the father and mother were strictly temperate, and yet, during the nine months previous to the birth of the child the mother uses spirituous liquors for any cause, just in proportion as she does so the child will inherit the appetite for strong drink. But if the mother is strictly temperate during the whole time previous to the child's birth, yet, while she nurses it, drinks ale, or beer, or porter, or spirits, " to make milk," or for any other cause, or gives the infant food or drink mixed with liquor, the child will be impregnated with the love of it. Thus it is that the surroundings of the mother, during gestation and nursing, impress upon the child its physical and moral character ; hence the regeneration of the race must come from maternal influence, hence the hopes of mankind for the amelioration of the condition of society in the future, its improvement in the physical constitution, in mental vigor and moral power, are founded in the proper education of our daughters for maternal and domestic duties, and a higher appreciation of their vast responsibilities in the directions above suggested. On the other hand, man comes in for his share in the great work, as father and husband, by giving his cordial coöperation to the same great end by all the means possible to him in labor, self-denial and generous sympathies.

